# Introduction to the Special Issue: Legumes as Food Ingredient: Characterization, Processing, and Applications

**DOI:** 10.3390/foods9111525

**Published:** 2020-10-23

**Authors:** Alfonso Clemente, Jose C. Jimenez-Lopez

**Affiliations:** Estación Experimental del Zaidín (CSIC), Consejo Superior de Investigaciones Científicas (CSIC), 18008 Granada, Spain; josecarlos.jimenez@eez.csic.es

**Keywords:** legumes, pulses, nutritional properties, health benefits, processing, microbiota, allergens, fermentation, sensory properties

## Abstract

Legumes are major ingredients in the Mediterranean diet, playing an essential role in developing countries. Grain legumes, such as lentil, chickpea, pea, lupin and beans, among others, are recognized as good sources of proteins, starch, fiber, vitamins and minerals for human nutrition, being an essential food crop for people worldwide. Due to their nutritional and techno-functional properties, legumes are widely used by the food industry as ingredients in a wide range of products for general and specific groups of the population, including vegetarians, diabetics or celiac patients. The Special Issue “Legumes as Food Ingredients: Characterization, Processing, and Applications” covers key aspects regarding the nutritional quality of legume flours and their derived products, as well as the health benefits of some of their bioactive components. The amounts of antinutritional components, such as certain allergens that might pose risks to sensitized consumers, are reported to be reduced by processing. Several pretreatments, including fermentation with lactic bacteria and yeasts, are used to improve the nutritional and sensory profile of the legume-derived products, increasing their acceptance by consumers.

Grain legumes and their derived products are increasingly used in food industry due to their nutritional composition and technological properties. The presence of bioactive compounds in legume seeds has been reported to exert beneficial effects in terms of tackling chronic disorders, including diabetes, cardiovascular diseases, and inflammatory and carcinogenic processes. As a result, Governmental and Health Agencies recommend as a healthy habit the regular consumption of legumes. These are largely used as ingredients by the food industry, being employed in a wide range of processes, including heat treatment, roasting, milling, canning, germination and fermentation, among others, that might influence their nutritional and sensory profile. On this issue, nine research papers and one review are published in this Special Issue.

Marin-Manzano et al. [1] reported the prebiotic properties of non-fructosylated α-galactooligosaccharides from pea seeds in infants. These oligosaccharides were fully fermented by fecal microbiota, lactic acid and short chain fatty acids were generated, and strong bifidogenic activity was observed. As a result, its potential use has been suggested as a prebiotic in infant formula.

The presence of allergen traces in foodstuffs might elicit allergic reactions in sensitized consumers. Regarding this, a sensitive and accurate ELISA methodology to detect, identify and quantify the lupin major allergen β-conglutin (Lup an 1) in raw and processed (roasted, fermented, boiled, cooked, pickled, toasted, pasteurized) lupin-derived foods was developed [2]. This assay might be considered as a reliable method to be used for the detection of lupin in a range of food matrices. In a comparative study, the levels of eight major soybean allergens in genetically and non-genetically modified soybeans were evaluated using ELISA and immunoblotting techniques; the overall data indicated that genetically modified soybeans did not show significant differences in the levels of the soybean allergens compared to non-genetically modified soybeans [3]. Lentil seeds are a source of high-quality proteins, being increasingly used in food industry as ingredients in a wide range of food formulations. Khazaei et al. [4] reviewed the current knowledge on lentil proteins and their bioactive peptides, the methods used for their extraction and isolation, and their potential applications in the food industry. 

The nutritional and sensory profiles of four underutilized *Acacia* species have been reported [5]. Their nutritional value suggests their potential to be included in the human diet or used in food formulations. Khrisanapant et al. [6] reported the characterization of volatile (aldehydes, alcohols, ketones, esters, terpens and hydrocarbons) and fatty acid profiles for up to 11 legume species, identifying some compounds that are specific for certain legume species. This information could be used by plant breeders and the food industry for the elaboration of legume products with specific volatile profiles. 

The fermentation of legume flours is carried out by the food industry for the improvement of the nutritional, functional or sensory properties of legume products. Thus, the lactic acid fermentation of faba bean flours has been demonstrated as a potential pretreatment for improvement of the nutritional quality of gluten-free faba breads, without affecting their sensory properties [7]. Schlegel et al. [8] reported the fermentation of lupin protein isolated with up to eight different microorganisms, and the effects on the sensory and techno-functional (protein solubility, foaming and emulsifying capacity) properties, and the protein’s resistance to the fermentative process, were both evaluated. The fermentation of a pea protein solution with a starter culture of lactic bacteria and different yeasts significantly reduced the presence of green-off compounds; such results are of interest for the food industry in terms of increasing the acceptability of plant matrices to consumers [9].

Finally, Kim et al. [10] reported the use of superfine defatted soybean flour obtained by jet milling for the preparation of fiber-enriched tofu, without affecting the physical and sensory properties of the product. 
